# Epidemiological study of accidents with biological material involving healthcare workers exposed to hepatitis B, C and HIV

**DOI:** 10.1186/1753-6561-5-S6-P224

**Published:** 2011-06-29

**Authors:** CT Zogheib, MM Baraldi, MM Simonetti, CM Santoro

**Affiliations:** 1 Serviço de Controle de Infecção Hospitalar, Hospital Alemão Oswaldo Cruz, São Paulo, Brazil

## Introduction / objectives

Health professionals are often at risk of accidents during the caution, and that these exposures may occur to the transmission of pathogens such as human immunodeficiency virus (HIV), Hepatitis B and Hepatitis C. The Committee on Infection Control has a fundamental role in the performance of the first visit to the accident, through guidance as to the serological monitoring and adherence to the monitoring of employees.

## Methods

In this study in a general hospital, were analyzed all accidents involving biological material in the period 2003 to 2010, by attending at the time of the accident, with research into the causes and serological employees exposed during the accident involving biological material and collection of serologic of source patients.

## Results

During the period, 398 accidents were monitored, and 234 (59%) with negative source patients, eight (2%) with the source patient Hepatitis B, 42 (10%) source patients with hepatitis C, 20 (5 %) source patients with HIV and 90 (23%) patients with unknown source, which enables these patients have positive tests questioned. During the first three visits were diagnosed (0.75%) employees were seropositive for hepatitis C and no history of accidents with biological material. Of these, 16 (4%) did not complete the follow-up due to the shutdown of the institution, despite subsequent contact. E 382 (96%) employees did not show seroconversion.

## Conclusion

Adherence to institutional guidelines for sharps injuries and the traceability of the source, the introduction of chemoprophylaxis, immunization against hepatitis B, are shown to be key measures in the management of accidents with biological material.

## Disclosure of interest

None declared.

